# MicroRNA-375 plays a dual role in prostate carcinogenesis

**DOI:** 10.1186/s13148-015-0076-2

**Published:** 2015-04-10

**Authors:** Pedro Costa-Pinheiro, João Ramalho-Carvalho, Filipa Quintela Vieira, Jorge Torres-Ferreira, Jorge Oliveira, Céline S Gonçalves, Bruno M Costa, Rui Henrique, Carmen Jerónimo

**Affiliations:** Cancer Biology and Epigenetics Group - Research Center (Lab3), Portuguese Oncology Institute - Porto (IPO-Porto), Rua Dr. António Bernardino de Almeida, Porto, 4200-072 Portugal; School of Allied Health Sciences (ESTSP), Polytechnic of Porto, Rua Valente Perfeito 322, Vila Nova de Gaia, 4400-330 Portugal; Department of Urology, Portuguese Oncology Institute, Rua Dr. António Bernardino de Almeida, Porto, 4200-072 Portugal; Department of Pathology, Portuguese Oncology Institute - Porto, Rua Dr. António Bernardino de Almeida, Porto, 4200-072 Portugal; Life and Health Sciences Research Institute (ICVS), School of Health Sciences, University of Minho, Rua da Universidade, 4704-553 Braga, Portugal; ICVS/3B’s - PT Government Associate Laboratory, Universidade do Minho, Campus de Gualtar, 4710-057 Guimarães, Braga Portugal; Department of Pathology and Molecular Immunology, Institute of Biomedical Sciences Abel Salazar (ICBAS), University of Porto, Rua de Jorge Viterbo Ferreira 228, Porto, 4050-313 Portugal

**Keywords:** Prostate cancer, MicroRNAs, Epigenetics, miR-375, *CCND2*

## Abstract

**Background:**

Prostate cancer (PCa), a highly incident and heterogeneous malignancy, mostly affects men from developed countries. Increased knowledge of the biological mechanisms underlying PCa onset and progression are critical for improved clinical management. MicroRNAs (miRNAs) deregulation is common in human cancers, and understanding how it impacts in PCa is of major importance. MiRNAs are mostly downregulated in cancer, although some are overexpressed, playing a critical role in tumor initiation and progression. We aimed to identify miRNAs overexpressed in PCa and subsequently determine its impact in tumorigenesis.

**Results:**

MicroRNA expression profiling in primary PCa and morphological normal prostate (MNPT) tissues identified 17 miRNAs significantly overexpressed in PCa. Expression of three miRNAs, not previously associated with PCa, was subsequently assessed in large independent sets of primary tumors, in which miR-182 and miR-375 were validated, but not miR-32. Significantly higher expression levels of miR-375 were depicted in patients with higher Gleason score and more advanced pathological stage, as well as with regional lymph nodes metastases. Forced expression of miR-375 in PC-3 cells, which display the lowest miR-375 levels among PCa cell lines, increased apoptosis and reduced invasion ability and cell viability. Intriguingly, in 22Rv1 cells, which displayed the highest miR-375 expression, knockdown experiments also attenuated the malignant phenotype. Gene ontology analysis implicated miR-375 in several key pathways deregulated in PCa, including cell cycle and cell differentiation. Moreover, *CCND2* was identified as putative miR-375 target in PCa, confirmed by luciferase assay.

**Conclusions:**

A dual role for miR-375 in prostate cancer progression is suggested, highlighting the importance of cellular context on microRNA targeting.

**Electronic supplementary material:**

The online version of this article (doi:10.1186/s13148-015-0076-2) contains supplementary material, which is available to authorized users.

## Background

Prostate cancer (PCa), the second most incident cancer in men worldwide (31.1%) [1] and ranking first in incidence in the US (27%) [2], is very heterogeneous, ranging from clinical indolent to extremely aggressive disease, causing substantial morbidity and mortality [3]. Adequate management is, thus, mandatory to avoid overtreatment, on one hand, and sub-optimal therapy, on the other. A better understanding of the biological mechanisms underlying PCa onset and progression are likely to contribute to improved clinical and therapeutic management.

Over the last two decades, deregulation of epigenetic mechanisms has emerged as a relevant driving force in PCa [4], with recent emphasis in altered microRNAs (miRNAs) expression [5]. MicroRNAs are a class of small non-coding RNAs, approximately 22 nucleotides in length [6], highly conserved along the evolutionary chain, with tissue and developmental stage-specific expression [7]. Currently, more than 2,000 human miRNAs are registered in the miRBase [8] and thought to negatively regulate gene translation, thus decreasing gene expression, and have been extensively implicated in several crucial cellular pathways, such as apoptosis, differentiation, and proliferation [9]. Interestingly, a single miRNA might have multiple targets, and a single mRNA may be targeted by several miRNAs [10].

In cancer, miRNAs are globally downregulated, although some are notoriously upregulated [11]. Such trend has been found in PCa, but most studies limit their analysis to expression array results, based on few tumor samples, and generally lacking subsequent validation in larger and independent datasets [5,12]. This may, at the least partially, explain contradictory results in the literature, making it difficult to establish specific PCa miRNA signatures [13]. Thus, assessing miRNAs differential expression in a robust cohort of patients carrying primary tumors and searching for miRNAs targets are critical to investigate its relevance in PCa initiation and progression.

In this study, we sought to discover miRNAs upregulated in PCa and unveil its role in prostate carcinogenesis through modulation of miRNAs expression and identification of putative molecular targets. Using a customized, commercially available, platform, a small set of miRNAs upregulated in PCa was identified, some of which have been previously reported. Validation in two large independent sets of patients demonstrated that miR-375 expression was increased in PCa with higher Gleason score and more advanced pathological stage, which entail worse prognosis. Following validation in clinical samples, miR-375 was also found to be upregulated in PCa cell lines compared to RWPE-1 (a benign prostate epithelial cell line). Modulation of miR-375 expression in two PCa cell lines (22Rv1 and PC-3) showed that this miRNA is involved in regulation of cell viability and apoptosis, in a cell-context-dependent manner. Furthermore, using a custom gene panel to search for potential targets followed by specific luciferase assay validation, *CCND2* was identified and confirmed as miR-375 target in PCa. Our observations thus suggest that miR-375 overexpression may contribute to prostate carcinogenesis and disease progression.

## Results

### MicroRNAs expression in prostate cancer tissues

**Figure 1:**
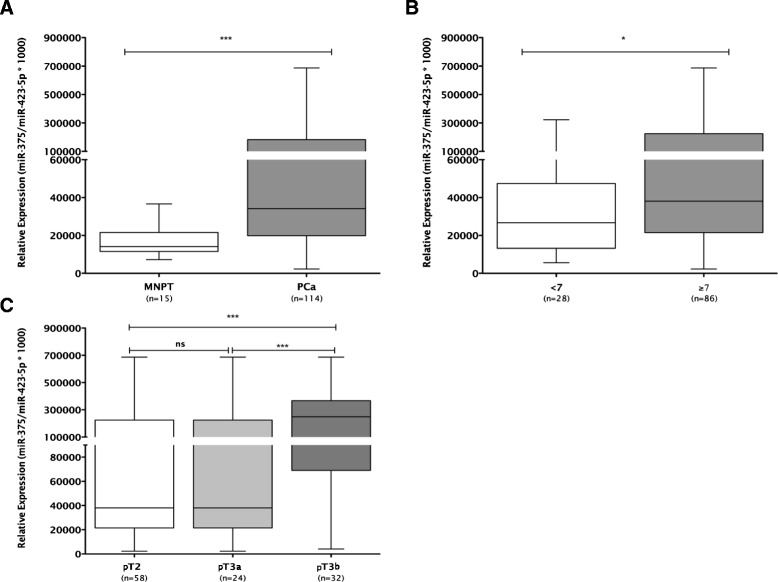
Expression levels of miR-375 in PCa and normal prostate tissues **(A)**, according to Gleason Score **(B)** and pathologic stage **(C)**.***P < 0.001; *P < 0.05.

Global expression of miRNAs was initially assessed and compared in ten PCa and four morphological normal prostate (MNPT) samples. Global miRNAs downregulation was found in PCa, although overexpression, with fold variation higher than 1.5, was depicted for 17 miRNAs (Table 1). Among these, those previously associated with PCa in the literature were excluded from further analysis, and the remainders were further selected for validation in a large sample set, and the respective clinical and pathological characteristics are provided in Table 2 (Additional file 1: Table S1). No significant differences in age were apparent between the two groups. Because several miRNAs were below detection level in quantitative reverse transcription-polymerase chain reaction (RT-qPCR) analyses (probably due to low expression levels), only three miRNAs (miR-32, miR-182, and miR-375) were assessed in the larger dataset. Whereas miR-182 and miR-375 were significantly overexpressed in PCa (*P* < 0.001 for both), confirming the results of the array, no significant differences were found for miR-32 expression between PCa and MNPT (Figure 1A and Additional file 2: Figure S1). MiR-375 expression levels were significantly higher in cases with higher Gleason score and more advanced pathological stage at diagnosis (*P* < 0.05, *P* < 0.001, Figure 1B, C).

**Table 1:** **List of overexpressed microRNAs in low-density miRNA RT-qPCR analysis (fold variation represents median values for PCa** ***vs*** **. MNPT)**

**MicroRNA**	**Fold variation (PCa** ***vs*** **. MNPT)**
miR-449a#	3.92
*miR-32*	*3.49*
miR-548c-5p	2.71
miR-562	2.56
miR-103-as	2.53
miR-512-3p	2.41
miR-200c*	2.33
miR-147b	2.24
miR-770-5p	2.09
miR-518c*	2.00
miR-517b	1.88
*miR-182*	*1.79*
miR-615-3p	1.70
miR-496	1.59
miR-1200	1.58
*miR-375*	*1.54*
miR-551a	1.53

*Passanger strand. #Already studied in other work by our research group. MNPT, morphological normal prostate; PCa, prostate cancer. In italics are the microRNAs chosen for further validation.

**Table 2:** **Clinical and pathological data of patients included in miR-375 validation**

**Clinicopathological data**	**Tumors (** ***n*** **= 114)**	**MNPT (** ***n*** **= 15)**
Age (years), median (range)	65 (49 to 74)	64 (45 to 80)
PSA (ng/mL), median (range)	8.00 (2.66 to 20.00)	n.a.
Pathological stage, *n* (%)		
pT2	58 (38.7%)	n.a.
pT3a	24 (16.0%)	n.a.
pT3b	33 (21.3%)	n.a.
Gleason score, *n* (%)		
<7	28 (18.7%)	n.a.
=7	73 (48.7%)	n.a.
>7	13 (8.7%)	n.a.

n.a., not applicable; MNPT, morphological normal prostate.

### Meta-analysis of miRNA expression in prostate cancer patients from The Cancer Genome Atlas

**Figure 2:**
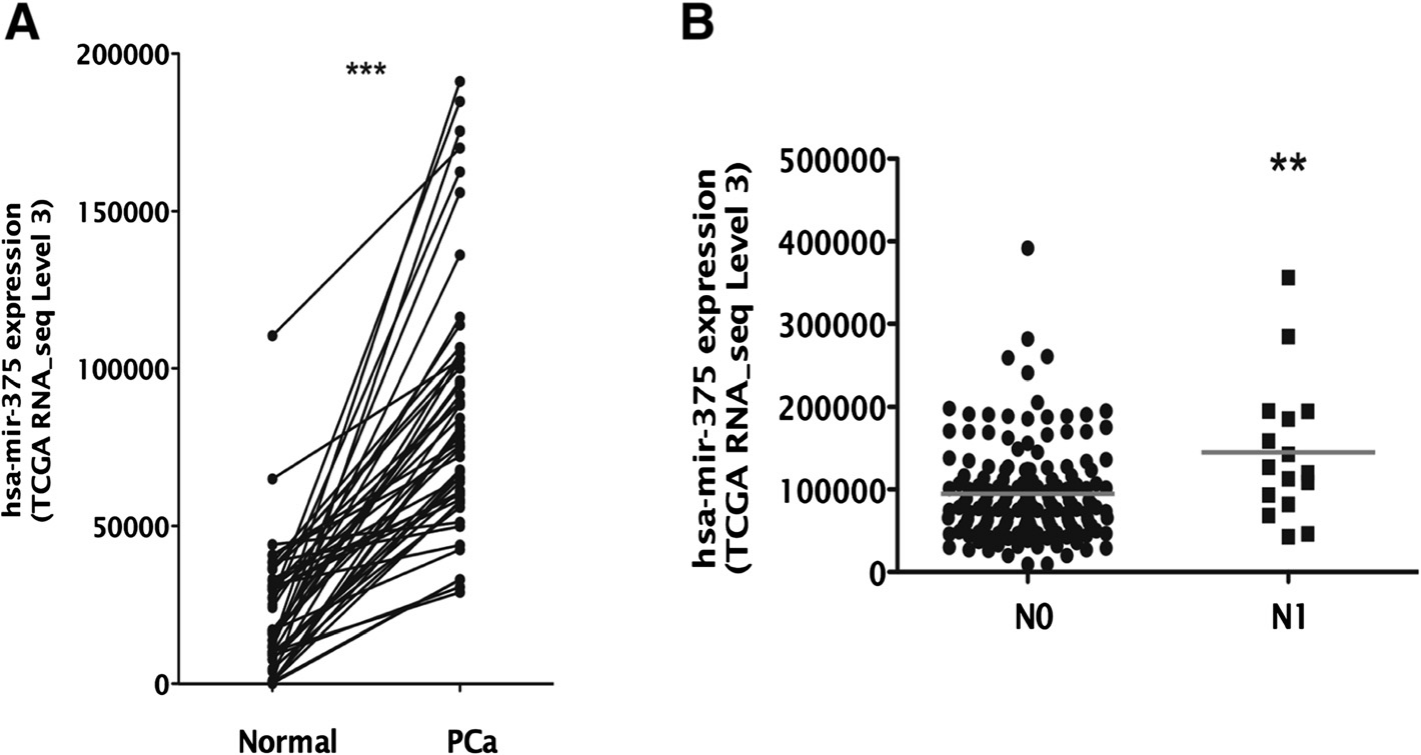
Expression of has-mir-375 is increased in prostate cancer and associates with lymph node stage in patients from TCGA. **(A)** miR-375 expression (TCGA miRNAseq RPKM level 3 value) was evaluated in 50 tumor and matched normal samples of prostate adenocarcinoma patients from TCGA. All tumor samples presented overexpression comparing to their matched normal samples (***P < 0.0001). **(B)** The expression of hsa-mir-375 (TCGA miRNAseq RPKM level 3 value) was assessed in 177 prostate adenocarcinoma patients from TCGA with lymph node stage information available. Patients in the N1 lymph node stage presented significantly higher expression of hsa-mir-375 than patients in the N0 stage.

Further validation of the array results was performed in a larger and independent dataset, that is, the miRNAseq expression data from PCa patients and matched normal samples deposited in The Cancer Genome Atlas (TCGA) (*n* = 326 and *n* = 50, respectively). Strikingly, miR-375 was significantly overexpressed in all tumors compared to matched normal samples (*P* < 0.0001; Figure 2A), as well as in patients with regional lymph node metastasis (N1) compared to those without regional lymph node involvement (N0) (*P* = 0.0017; Figure 2B). Moreover, both miR-32 and miR-182 were overexpressed in PCa compared to matched normal prostate tissues (*P* < 0.0001; Additional file 3: Figure S4).

**Figure 3:**
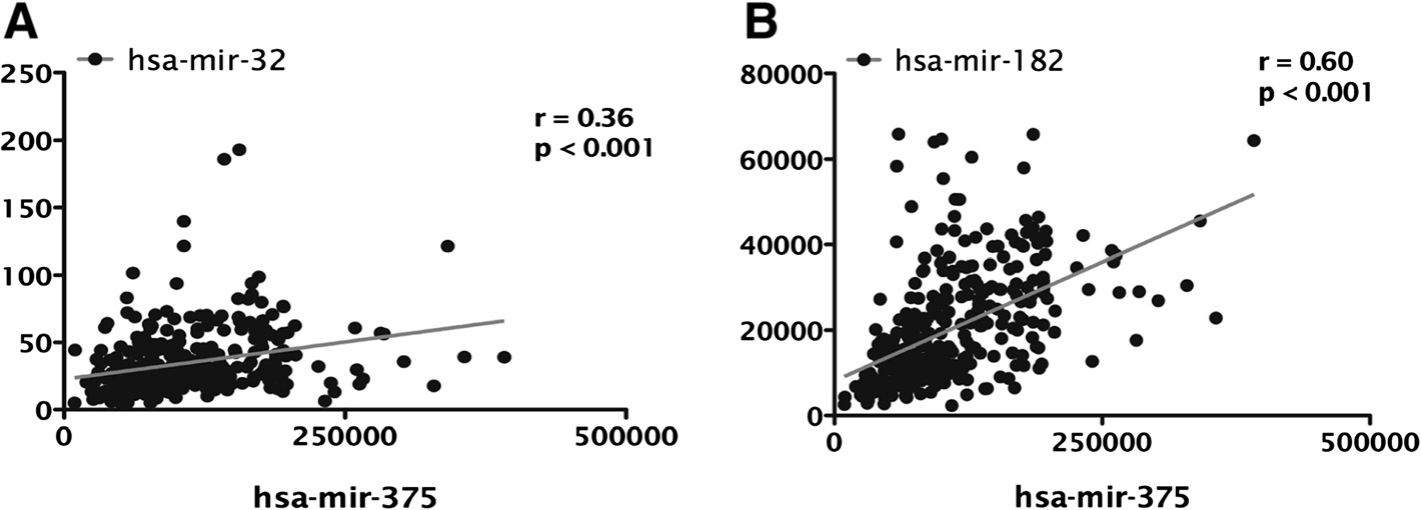
miR-375 is significantly co-expressed with **(A)** miR-32 and **(B)** miR-182 in prostate cancer patients from TCGA (*r* calculated by Spearman’s correlations).

Correlation analysis for miRNAs expression showed that miR-375 was significantly co-expressed with miR-32 and miR-182 (*r* = 0.36 and *r* = 0.60, respectively; Table 3 and Figure 3).

**Table 3:** **Validation of the correlation between hsa-mir-375 and identified upregulated microRNAs in prostate adenocarcinoma samples from TCGA dataset** [40]

**microRNA**	**Spearman’s correlation**	***P*** **value**
hsa-mir-449a	0.27351	<0.0001
hsa-mir-32	0.36431	<0.0001
hsa-mir-548c	N/A	
hsa-mir-562	N/A	
hsa-mir-103-1-as	N/A	
hsa-mir-103-2-as	N/A	
hsa-mir-512-1	0.13177	0.01729
hsa-mir-512-2	0.05064	0.36211
hsa-mir-200c	0.59926	<0.0001
hsa-mir-147b	0.12526	0.02370
hsa-mir-770	−0.09100	0.10099
hsa-mir-518c	−0.01563	0.77866
hsa-mir-517b	−0.03261	0.55740
hsa-mir-182	0.60472	<0.0001
hsa-mir-615	0.39231	<0.0001
hsa-mir-496	−0.23667	<0.0001
hsa-mir-1200	N/A	
hsa-mir-551a	−0.00138	0.98014

N/A, not applicable.

### MiR-375 expression in prostate cell lines

**Figure 4:**
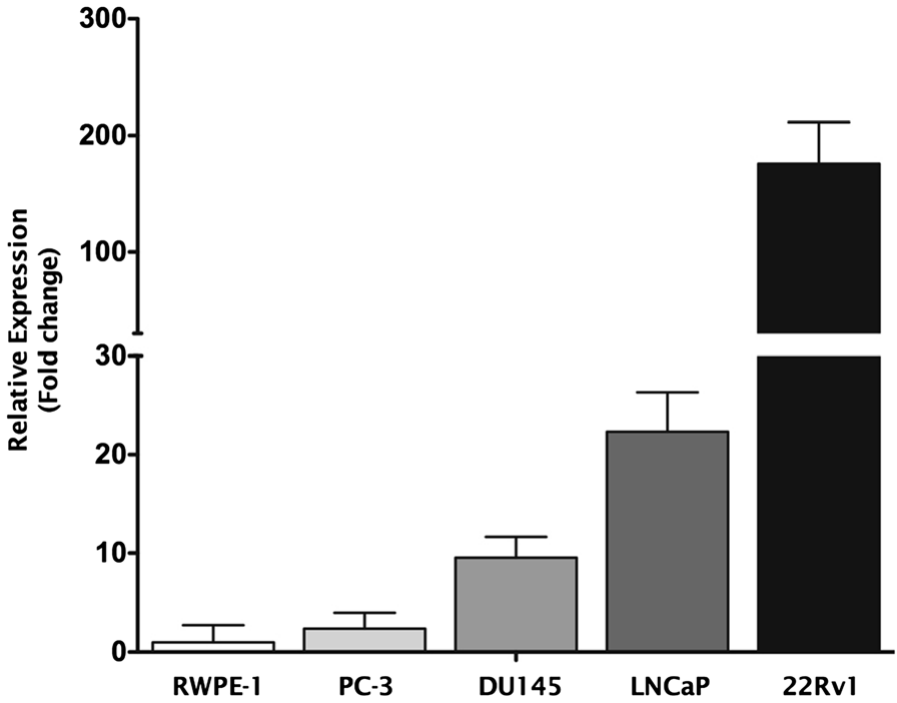
miR-375 expression levels in prostate cell lines. Results are displayed after normalization to RWPE-1.

PCa cell line 22Rv1 depicted the highest miR-375 expression levels, whereas the lowest were found in PC-3 cells (Figure 4). In RWPE-1 cells, miR-375 expression levels were lower than those of any PCa cell line, mimicking the results of PCa and MNPT tissues. Thus, those cell lines were selected for subsequent functional experiments of miR-375 downregulation (22Rv1) or forced expression (PC-3 and RPWE-1).

### Phenotypic impact of miR-375 forced expression in PC-3 cells and RWPE-1 cells

**Figure 5:**
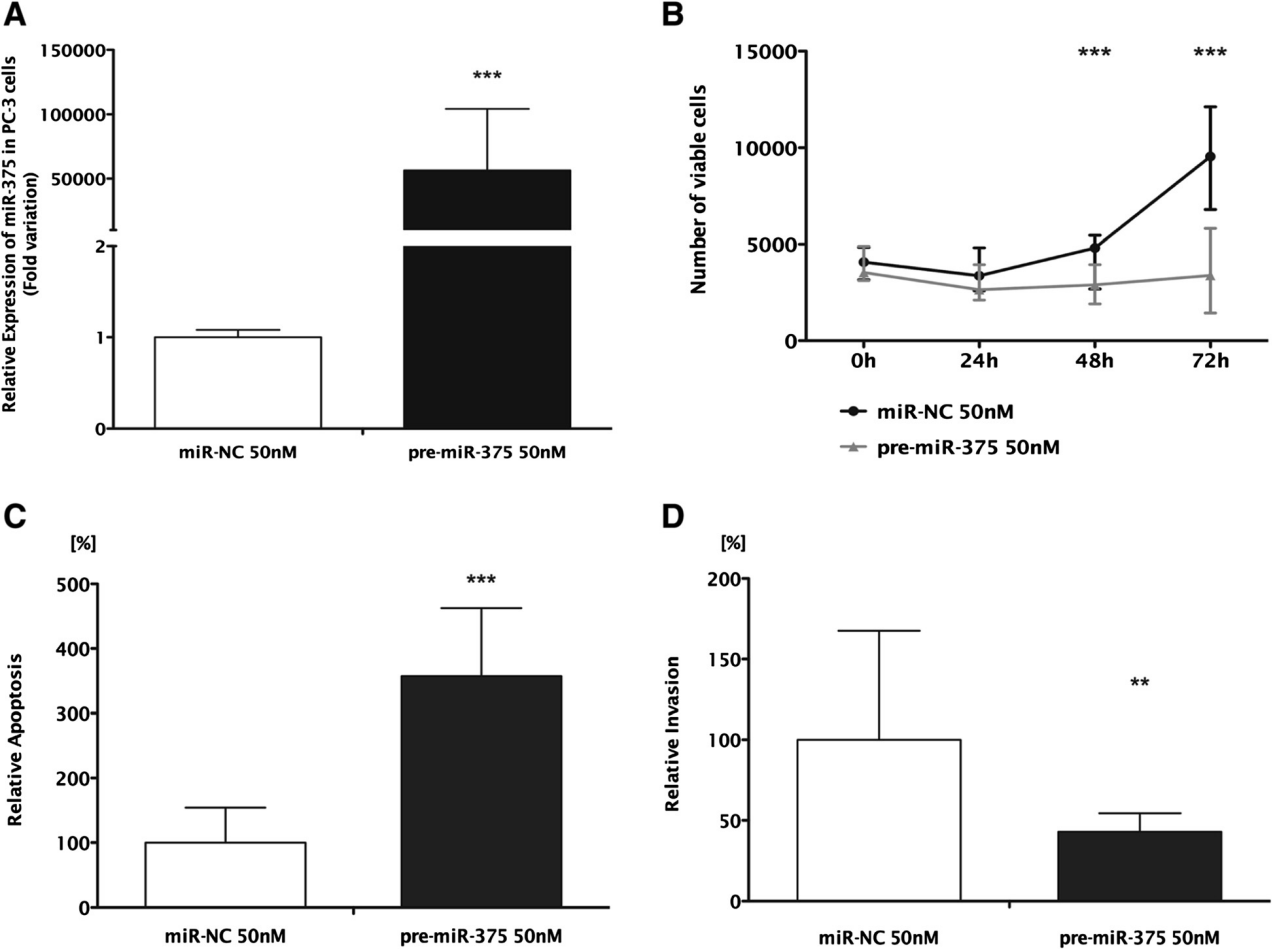
Forced expression of miR-375 in PC-3. **(A)** Relative expression of miR-375 (normalized to miR-NC), **(B)** number of viable cells, **(C)** relative apoptosis levels (normalized to miR-NC), and **(D)** relative invasion (normalized to miR-NC). **(B**, **C**, **D)** Data shown as the median of three independent experiments and range performed in triplicate (***P < 0.001; **P < 0.01).

**Figure 6:**
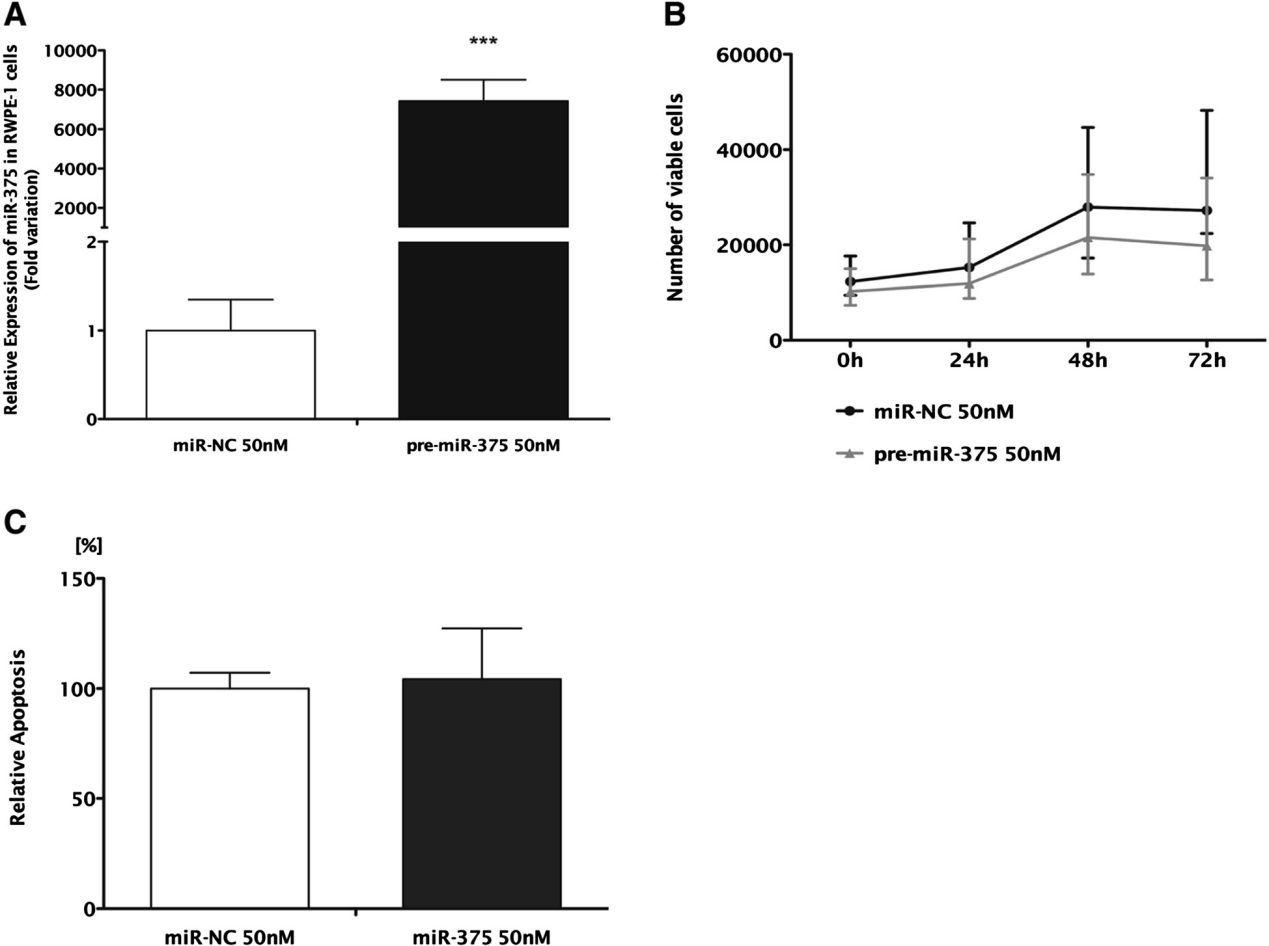
Forced expression of miR-375 in RWPE-1. **(A)** Relative expression of miR-375 (normalized to miR-NC), **(B)** number of viable cells, and **(C)** relative apoptosis levels (normalized to miR-NC). **(B**, **C)** Data shown as the median and range of three independent experiments performed in triplicates. ***P < 0.001.

At 72 h after transfection, miR-375 expression levels were increased 56,000 and 8,000 times in PC-3 and RPWE-1 cells, respectively (*P* < 0.001 for both) (Figures 5A and 6A). PC-3 cells’ viability was significantly reduced at 48 h (39%, *P* < 0.01) and 72 h (60.5%, *P* < 0.001) (Figure 5B). Moreover, 72 h after transfection, apoptosis levels were significantly increased (more than threefold (*P* < 0.001), Figure 5C). Because PC-3 is highly invasive, this feature was evaluated following miR-375 forced expression and a maximum of 52% reduction was observed in transfected cells (*P* < 0.01) (Figure 5D and Additional file 4: Figure S2). In transfected RWPE-1 cells, and despite a significant increase in miR-375 levels, no significant alterations in cell viability and apoptosis were apparent (Figure 6B, C), suggesting that miR-375 deregulation is mostly relevant in the context of malignant prostate cell.

### Phenotypic impact of miR-375 downregulation in 22Rv1 cells

**Figure 7:**
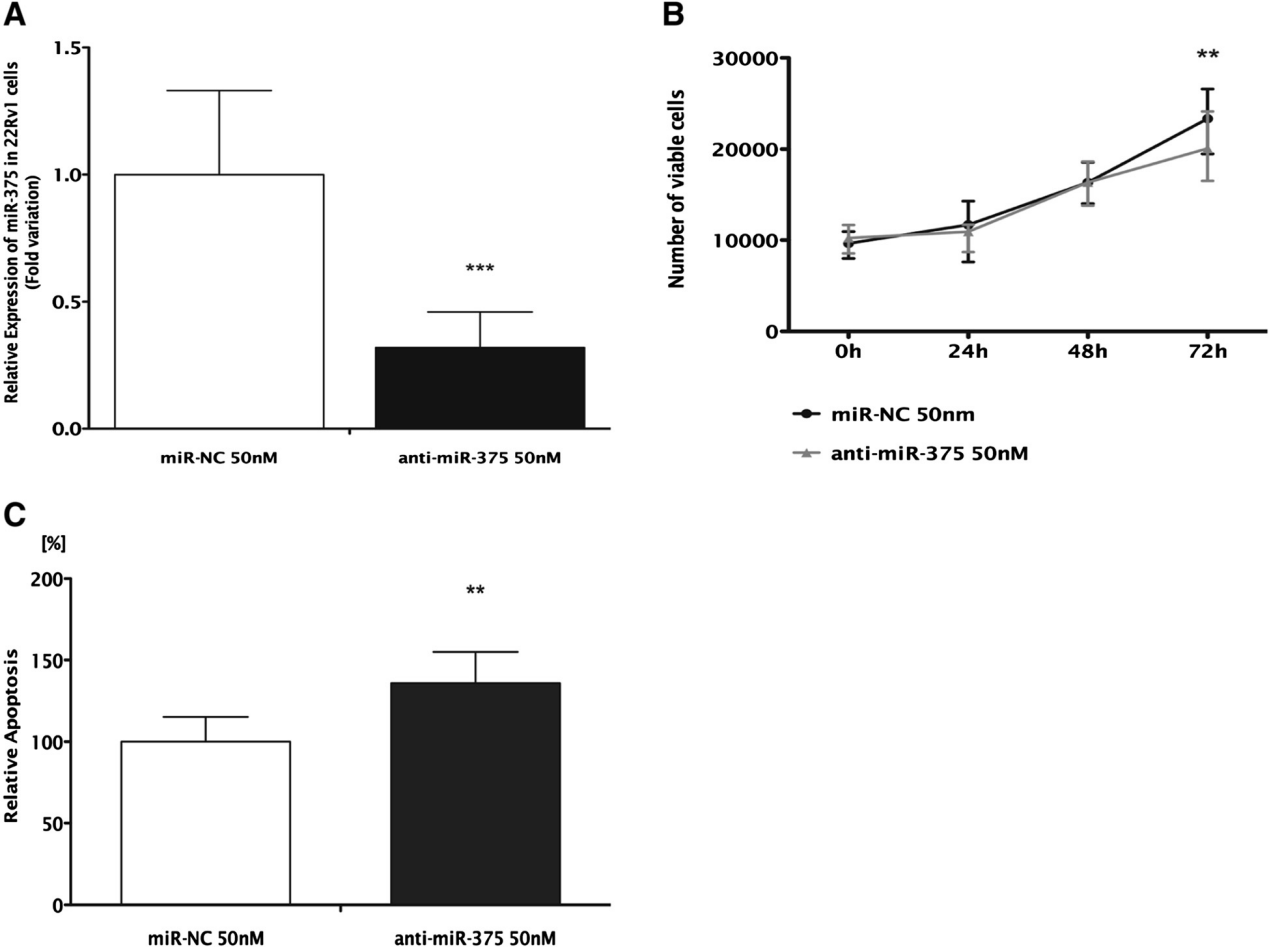
Inhibition of miR-375 in 22Rv1. **(A)** Relative expression of miR-375 (normalized to miR-NC), **(B)** number of viable cells, and **(C)** relative apoptosis levels (normalized to miR-NC). **(B**, **C)** Data shown as the median and range of three independent experiments performed in triplicates (***P < 0.001; **P < 0.01).

Expression levels of miR-375 were decreased by 68% 72 h after transfection (*P* < 0.001; Figure 7A) and this was associated with significantly reduced cell viability (*P* < 0.01). At this time point, viable cells reached only 17% (Figure 7B) and apoptosis increased by 30% (*P* < 0.01) in 22Rv1-transfected cells (Figure 7C). Because this cell line displays low invasive ability, invasion assay was not performed.

### Putative miR-375 targets

**Figure 8:**
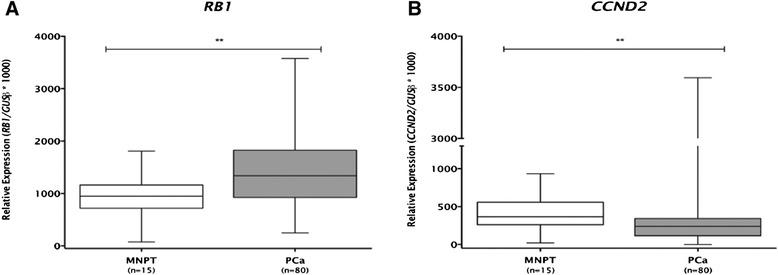
Expression levels of potential miR-375 targets for array validation **(A)** *RB1* and **(B)** *CCND2* (**P < 0.01).

**Figure 9:**
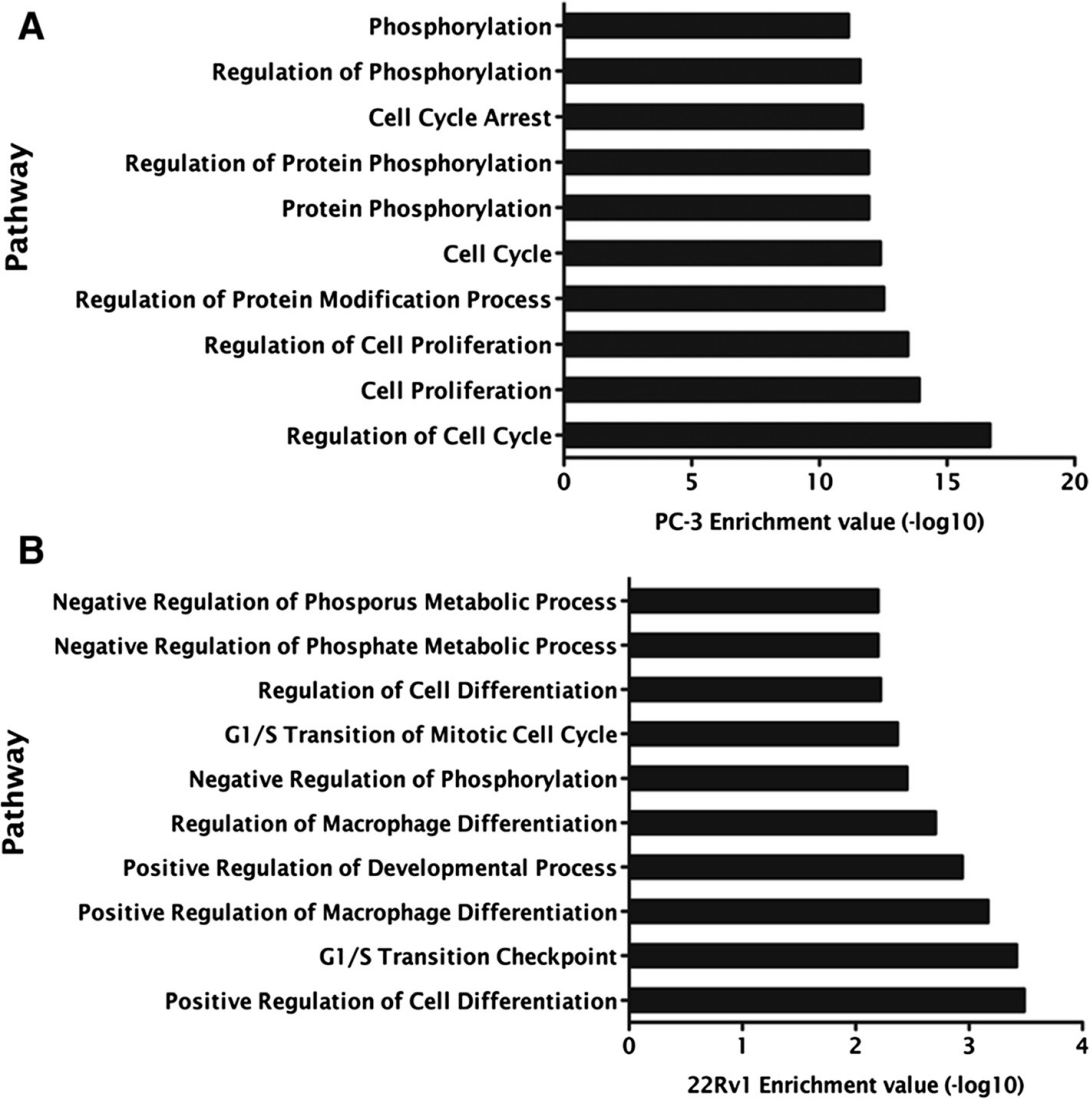
GO enrichment analysis: pathways **(A)** downregulated in PC-3 and **(B)** upregulated in 22Rv1 transfected cells.

The search for putative miR-375 targets comprised expression analysis of 61 genes implicated in some of the most important cellular pathways deregulated in cancer. Thus, expression profiles of PC-3 transfected (pre-miR-375) and 22Rv1 transfected (anti-miR-375) cells were compared to its respective controls (Additional file 5: Figure S3). In 22Rv1 cell with miR-375 downregulation, *RB1* was upregulated, whereas in miR-375-overexpressing PC-3 cells, *CCND2* was downregulated (*P* < 0.001 for both). These findings were validated in tissue samples as *RB1* transcript levels were significantly increase and *CCND2* expression levels were significantly decreased in primary PCa compared to MNPT (Figure 8). Furthermore, gene ontology enrichment analysis disclosed that genes involved in cell cycle regulation were those more frequently deregulated in 22Rv1 and PC-3 cell lines (Figure 9), supporting a key role for miR-375 in PCa.

### miR-375 directly targets *CCND2*

**Figure 10:**
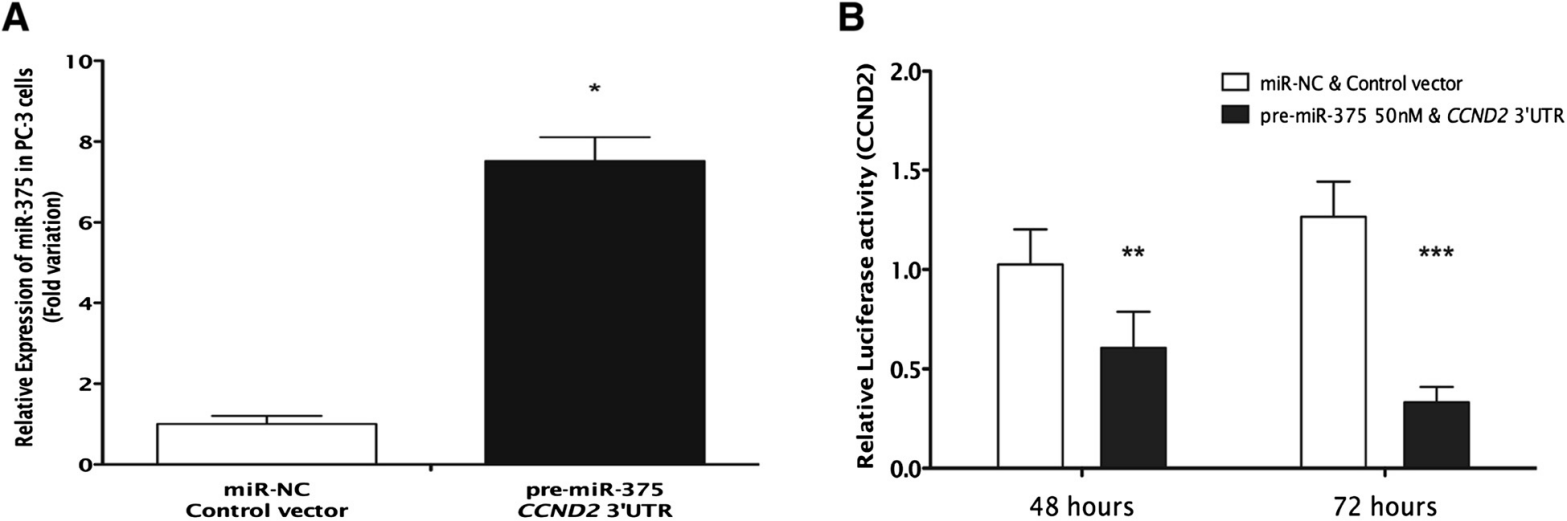
Luciferase reporter assay for the 3′UTR of *CCND2* in PC-3 cells. **(A)** Transfection of pre-miR-375 resulted in an increase of miR-375 upon transfection. **(B)** Luciferase activity was measured upon 48 and 72 h upon co-transfection of control vector with miR-NC and *CCND2* with pre-miR-375. Data shown as the median and range of three independent experiments performed in triplicates (***P < 0.001; **P < 0.01).

*In silico* analysis identified a miR-375 potential binding site at *CCND2* 3′untranslated region (UTR). Moreover, analysis of TCGA data disclosed a statistically significant negative correlation between miR-375 and *CCND2* expression in PCa tissues (Spearman’s correlation, *r* = −0.57, *P* < 0.0001). Luciferase assay was performed in PC-3 cells to determine whether miR-375 might regulate *CCND2* transcription levels. In *CCND2* 3′UTR vector and pre-miR-375 co-transfected PC-3 cells, a sixfold increase in miR-375 expression levels was apparent at 72 h (Figure 10A), whereas CCND2 3′UTR luciferase activity was 37% reduced, at 48 h (*P* < 0.01), and 71% at 72 h (*P* < 0.001) after transfection (Figure 10B).

## Discussion

Prostate cancer remains a major challenge, mostly due to insufficient knowledge about the factors determining its onset and progression [14]. Because epigenetic alterations play an important role in prostate carcinogenesis and owing to the relative shortage of validated data on miRNA altered expression in PCa, we aimed to identify and validate miRNAs upregulated in this malignancy. Furthermore, its impact on malignant cell phenotype was assessed and putative target genes were identified. The strategy used in our study is similar to that of some previous publications on this subject [15,16].

Results of the miRNA expression array showed that, in PCa, miRNAs are mostly downregulated, whereas only a minor subset is overexpressed, confirming previous reports [13]. Because expression array data may be biased owing to the (usually) small number of samples assessed [17], we decided to validate the miRs overexpressed in the array, and not previously associated with PCa, in two large and independent datasets. Importantly, miR-182 and miR-375 overexpression was confirmed in the validation datasets. However, miR-32 overexpression was not validated and expression of other miRNAs was found to be minimal or absent. These observations further emphasize the need of validation studies following expression array experiments, as well as possible technical limitations for miRNA analysis.

Because a report on miR-182 overexpression in PCa was published during the execution of this study [18], we then proceeded with miR-375 for further analysis. Although MiR-375 expression levels in body fluids had been previously proposed as diagnostic and prognostic PCa biomarkers [19-21], its biological role in prostate carcinogenesis has not been investigated before. We found that, in PCa tissues, miR-375 expression was higher than in normal prostate tissues, paralleling the results of body fluids analysis [19-21]. Furthermore, higher expression levels associated with higher Gleason score and more advanced pathological stage, two clinicopathological parameters that entail unfavorable prognosis. These observations were further validated in an independent dataset through meta-analysis of TCGA data available for miR-375 expression in prostate tissues. Interestingly, an association of higher miR-375 expression levels and regional lymph node metastases was depicted, providing additional confirmation of our results. Although miR-32 expression levels did not differ between PCa and MNPT in our dataset, the larger number of samples available at the TCGA demonstrated increased expression PCa compared to matched normal prostate tissues. It is noteworthy that normal samples differ not only in number (15 *vs*. 50) but also in its nature as we used MNPT from patients not carrying PCa whereas normal prostate tissues from TCGA correspond to matched samples from PCa patients.

Although this and other studies [19-21] implicate miR-375 upregulation in PCa, miR-375 has been mostly considered a tumor suppressor, namely in gastric, head and neck, pancreatic, and hepatocellular cancers [22-25]. Indeed, in gastric cancer, miR-375 forced expression increased apoptosis and reduced of cell viability *in vitro* [25], and janus kinase 2 (*JAK2*) was identified as a direct target [26]. It should be emphasized, however, that miR-375-altered expression reports are mostly based in microarray or validation platform analysis attempts to discriminate different tumor subgroups according to miRNAs expression, seldom providing biological clues to the role of miR-375 in cancer [19,20,27-29].

Expression analysis results of miR-375 in prostate cells lines parallel those of primary tissues, as the lowest levels were found in RWPE-1, a benign prostate cell line. However, whereas 22Rv1 cells displayed high expression levels, PC-3 cells disclosed significantly lower levels. These findings provided a unique opportunity to evaluate the biological role of miR-375 in PCa cells, using two opposing, yet complementary strategies. Intriguingly, both anti-miR-375 transfection in 22Rv1 cells (causing 68% reduction in miR-375 expression levels) and forced miR-375 expression in PC-3 cells attenuated the malignant phenotype, whereas in RPWE-1 cells, forced miR-375 expression did not cause significant phenotypic alterations. Thus, while in 22Rv1 cells an oncogenic role for miR-375 is suggested, a tumor-suppressive function is implied for PC-3 cells. Considering that miRNAs might play an oncogenic or tumor-suppressive role depending on the cellular context in different tumors [19,22], this observation would not be surprising, except for the fact that it occurred in the same tumor model. However, due to the widely acknowledged heterogeneity of PCa, it may be reasonable to assume that in different prostate cancers (herein represented by different cell lines) miR-375 could play antagonistic roles.

Considering the potential dual role of miR-375 in PCa, the expression of 61 cancer-related genes, involved in critical cellular pathways, was assessed in PC-3 and 22Rv1 transfected cells. The panel of altered genes was, indeed, different in each cell line, as it would be expected from the results of the phenotypic assays and the baseline expression levels of miR-375. These results might also be explained by the acknowledged ‘promiscuity’ of miRNAs, as a single miRNA may target several different gene transcripts in a time- and model-dependent manner [10,30]. In the expression analysis, retinoblastoma 1 (*RB1*) and cyclin D2 (*CCND2*) surfaced as the most deregulated genes in each cell line. Remarkably, *CCND2* expression was found to be decreased in primary PCa samples, consistent with a putative target of miR-375 (which is overexpressed in those samples), whereas *RB1* was overexpressed in the same set of primary tumors. The former result is in line with previous reports on *CCND2* downregulation in PCa [31,32] whereas the latter contradicts previous observations concerning *RB1* downregulation in PCa [33]. However, it should be emphasized that in addition to miR-375, other epigenetic and/or genetic mechanisms, eventually more relevant *in vivo*, might be involved in RB1 expression regulation in PCa. Furthermore, the luciferase assay confirmed that *CCND2* is a target of miR-375 as *CCND2* transcript levels were significantly reduced after miR-375 forced expression. Interestingly, *CCND2* is a key element in cell cycle regulation, and this pathway was found as the most relevant in which miR-375 was implicated, in gene ontology enrichment analysis of both transfected cell lines. It should be recalled, however, that downregulation of *CCND2* has also been associated with aberrant promoter methylation [31] and, thus, different epigenetic mechanisms may act in concert to accomplish *CCND2* silencing in PCa.

Our results thus suggest that miR-375 plays a dual role in PCa, acting either as an oncomiR or a tumor-suppressor miRNA, depending on the cellular context. It is noteworthy that PCa cell lines with the highest miR-375 expression levels are androgen-responsive (22Rv1 and LNCaP), whereas androgen-independent (DU145 and PC-3) cells display the lowest levels. Strikingly, these results are in line with previous reports that associate DNMT activity, promoter methylation of miR-375 and androgens [34]. Interestingly, although derived from metastasis, 22Rv1 and LNCaP cell lines display the miR-375 expression profile typical of primary PCa from both series analyzed, and these are also androgen responsive as they represent clinically localized, androgen-ablation therapy-naïve tumors. Thus, the cellular context in which miR-375 may act as oncomiR or tumor suppressor is likely to be conditioned by androgen receptor regulation. Examples of miRNAs that may act as tumor suppressors or oncomiRs, depending on the tumor model, are acknowledged (for example, cluster miR-191/425 in breast cancer depending on estrogen status) [35]. However, this seems to be a previously unrecognized event in which a miRNA may have that dual role in the same tumor model, depending on the stage of tumor progression and hormonal environment. It is tempting to speculate whether in the same tumor, along its progression, miR-375 may act initially as an oncomiR and later as a tumor suppressor, targeting different genes as disease progresses. The mechanisms underlying this putative alteration remain elusive but it could derive from the significant alteration of the gene expression landscape of PCa cells along disease progression [36].

## Conclusions

In conclusion, our data provides further insight into miRNAs overexpression in PCa, suggesting that miR-375 upregulation might be act as oncomiR at the initial steps of prostate carcinogenesis, a role that could be impaired as PCa progresses, probably due to the cumulative genetic and epigenetic alterations endured by cancer cells. We provide evidence that miR-375 deregulation disturbs several critical cellular pathways, especially cell cycle regulation, eventually through *CCND2* targeting, which may, at the least partially, explain the frequent downregulation of *CCND2* in primary PCa.

## Methods

### Patients and sample collection

Primary tumors from 119 patients harboring clinically localized prostate adenocarcinoma were prospectively collected after diagnosis and primary treatment with radical prostatectomy at Portuguese Oncology Institute, Porto, Portugal. A set of 15 MNPTs was collected from prostatic peripheral zone of bladder cancer patients submitted to cystoprostatectomy. All tissue specimens were promptly frozen immediately after surgery. Upon histological confirmation of tumor or normal prostate tissue, fresh-frozen tissue fragments were trimmed to enhance yield of target cells (>70%). Histological slides from formalin-fixed paraffin-embedded tissue fragments were also routinely obtained from the surgical specimens and assessed for Gleason score and TNM stage. Relevant clinical data was collected from clinical charts. Informed consent was obtained from all participants, according to institutional regulations. This study was approved by the institutional review board [Comissão de Ética para a Saúde-(CES-IPOPFG-EPE 205/2013)] of Portuguese Oncology Institute - Porto, Portugal.

### Cell culture

Human PCa cell lines available in our lab (LNCaP, 22Rv1, DU145 and PC-3) and a non-malignant prostate cell line (RWPE-1, kindly provided by Prof. Margarida Fardilha, University of Aveiro, Portugal) were used in this study. All cell lines were cultured using recommended medium supplemented with 10% of fetal bovine serum and 1% of penicillin-streptomycin (FBS; GIBCO, Invitrogen, Carlsbad, CA, USA) and maintained at 37°C and 5% CO_2_ in a humidified chamber. All cell lines were routinely tested for contamination by *Mycoplasma spp.* using a specific multiplex PCR (PCR Mycoplasma Detection Set, Clontech Laboratories Inc., Mountain View, CA, USA).

### Total RNA extraction

Total RNA from clinical samples and cell lines was obtained by suspension in TRIzol® reagent (Invitrogen, Carlsbad, CA, USA) and, after adding chloroform, total RNA was purified from the aqueous phase of TRIzol® extract using PureLink™ RNA Mini Kit (Invitrogen, Carlsbad, CA, USA) following the manufacturer’s recommendations. RNA concentration, purity, and integrity of samples were determined on a Nanodrop ND-1000 spectrophotometer (NanoDrop Technologies, Wilmington, DE, USA) and electrophoresis.

### MicroRNAs global expression

Global miRNAs expression was assessed in ten PCa and four MNPT using microRNA Ready-to-Use PCR Human Panel (I + II) v2.0 (Exiqon, Vedbaek, Denmark), consisting of 739 miRNAs in total. RNA samples were submitted to cDNA synthesis using miRCURY LNA™ Universal RT microRNA PCR (Exiqon, Vedbaek, Denmark) following manufacturer’s instructions. Briefly, for each sample, 4 μL of 5× reaction buffer, 9-μL nuclease-free water, 2 μL of enzyme mix, 1 μL of synthetic spike in, and 4 μL of previously concentration-adjusted RNA. Tubes were then vortexed gently, and reverse transcription was performed in Veriti® Thermal Cycler (Applied Biosystems, Foster City, CA, USA). Protocol consisted of incubation for 60 min at 42°C, followed by 5 min at 95°C. Global expression was performed in a LightCycler 480 Instrument (Roche Diagnostics, Manheim, Germany) according to the manufacturer’s conditions. Data was analyzed using GenEX software (MultiD Analyses AB, Göteburg, Sweden). Then, data were analyzed using the comparative Ct method [37]. Median value of reference genes was used for normalization, and miRNAs with fold change higher than 1.5 were classified as overexpressed in PCa compared to MNPT.

### Validation of microRNAs expression

cDNA was synthesized from 119 PCa, 15 MNPT and 5 prostate cell lines, using miRCURY LNA™ Universal RT microRNA PCR (Exiqon, Vedbaek, Denmark), following the manufacturer’s instructions, as described above. Samples were then eluted 80× in nuclease-free water. MiRNAs’ levels were evaluated using specific primers (microRNA LNA™ PCR primer set, Exiqon, Vedbaek, Denmark) according to the manufacturer’s recommendations. In each well, 4 μL of diluted cDNA were mixed with 1 μL of specific miRNAs qPCR primers (Exiqon, Vedbaek, Denmark), 2 μL of ROX reference dye (Invitrogen, Carlsbad, CA, USA) and 5 μL of SYBR® Green Master mix (Exiqon, Vedbaek, Denmark). Protocol consisted in a denaturation step at 95°C for 10 min, followed by 40 amplification cycles at 95°C for 10 s and 60°C for 1 min. As previously mentioned, melting curve analysis was also performed at the end of the procedure according to instrument’s manufacturer recommendations. Each 96-well plate included multiple non-template controls and serial dilutions (10×) of cDNA obtained from human prostate RNA (Ambion, Invitrogen, Carlsbad, CA, USA) was used to construct a standard curve for each plate. All experiments were run in triplicates in a 7500 Sequence Detection System (Applied Biosystems, Foster City, CA, USA). Considering the results from global analysis, it was decided to use the reference gene with less variation (miR-423-5p) among samples for normalization of validation data. Relative expression of miRNAs was determined as target gene mean quantity/reference gene mean quantity. Values were then multiplied by 1,000 for easier tabulation.

### MicroRNAs transient transfection

miR-375 was transiently transfected in PC-3 and RWPE-1 cells with a Pre-miR™ miRNA precursor (pre-miR-375, PM10327, Applied Biosystems, Foster City, CA, USA) and an Anti-miR™ miRNA inhibitor (anti-miR-375, AM10327, Applied Biosystems, Foster City, CA, USA) was transfected for 22Rv1 cells. A miRNA negative control was used as control in all experiments (miR-NC, AM17010, Applied Biosystems, Foster City, CA, USA). Cells were seeded under standard conditions in six-well and 96-well plates for 24 h before transfection, reaching 30% to 50% confluence. In these experiments, pre-miR-375, anti-miR-375, and miR-NC concentration was 50nM. Oligofectamine™ reagent (Invitrogen, Carlsbad, CA, USA) was used under conditions indicated by the manufacturer. Cells were then incubated at 37°C and 5% CO_2_ in a humidified chamber for 72 h upon transfection. At 72 h, forced expression or silencing of miR-375 were confirmed by RT-qPCR.

### Cell viability assay

To evaluate the impact of *in vitro* transfection of miR-375 in PCaer cell lines, 3-(4,5-dimethylthiazol-2-yl)-2,5-diphenyltetrazolium (MTT; Sigma-Aldrich, Schnelldorf, Germany) assay was performed in 96-well plates. Briefly, cells were incubated with 10% MTT at 5 mg/mL in a humidified chamber for 24, 48, and 72 h after transfection. Reaction was stopped by removal of MTT and addition of 100 μL DMSO (Sigma-Aldrich, Schnelldorf, Germany) per well. Finally, plates were shaken for 15 min for complete dissolution. Absorbance levels were measured using a microplate reader (Fluostar Omega, BMG Labtech, Offenburg, Germany) at 540 nm with background deduction at 630 nm. Number of viable cells was obtained using the following formula: (OD experiment × Mean number of cells at 0 h)/Mean OD at 0 h. Three biologically independent experiments were performed, comprising methodological triplicates for each experiment.

### Apoptosis assay

Apoptosis was assessed using the APOPercentage™ kit (Biocolor Ltd., Belfast, Northern Ireland, UK). Cell lines were seeded under the same conditions as described for MTT assay and, after 72 h incubation, apoptosis assay was performed according to the manufacturer’s instructions. Quantification of apoptosis was achieved by measuring the optical density of the released dye at 550 nm with background deduction at 620 nm using a FLUOstar Omega microplate reader. To normalize the OD obtained for the apoptosis assays relatively to cell number, OD of cell viability assay at 72 h was used. Results were expressed as ratio of transfected cells OD to miR-NC OD (set as 100%).

### Invasion assay

Invasion ability of PC-3 transfected cells was analyzed using BD Biocoat™ Matrigel Invasion Chambers (BD Biosciences, Franklin Lakes, NJ, USA) according to the manufacturer’s protocol. Briefly, cells were seeded and then transfected in six-well plates. After 48 h of transfection, cells were trypsinized and seeded in serum-free culture medium in Matrigel inserts and allowed to invade for 24 h at 37°C and 5% CO_2_ in a humidified chamber. Medium with serum was used as chemoattractant. Then, non-invasive cells were removed from the top of the membranes and cells that invaded were fixed with methanol and stained with DAPI. Invasive cells were manually counted in a fluorescence microscope, and results were displayed as a percentage of cells that crossed the membrane (invading cells) relative to miR-NC.

### Identification of potential miR-375 target genes

To determine whether miR-375 was implicated in regulation of selected genes involved in cell cycle, apoptosis, DNA repair, mTOR, or MAPK/ERK pathways, a custom array panel (Roche Applied Science, Manheim, Germany) was designed for quantification of selected gene expression. Total RNA was extracted from all cell lines using TRIzol® (Invitrogen, Carlsbad, CA, USA) according to the manufacturer’s instructions and cDNA synthesis was performed using Transcriptor High Fidelity cDNA Synthesis Kit (Roche, Manheim, Germany) according to the manufacturer’s instructions. Expression levels were determined by real-time PCR in a LightCycler 480 (Roche Diagnostics, Manheim, Germany) and the amount of mRNA was normalized using *GUSB*, *TFRC*, and *18S* as endogenous controls. The comparative Ct method [37] was used to calculate fold-difference in gene expression between mir-375 transfected cells and respective miR-NC.

### Gene ontology enrichment analysis

Gene ontology enrichment (GOE) analysis was performed to ascertain which biological processes are regulated by miR-375 in PCa cell lines. AmiGO database [38] was used, and statistical analysis was performed using R program based on hypergeometric distribution followed by Fisher’s exact test [39].

### Expression of potential target genes in clinical samples

Following gene selection, mRNA levels were confirmed in the same group of tissue samples previously indicated. A total of 300 ng was reverse transcribed and amplified using TransPlex® Whole Transcriptome Amplification Kit (Sigma-Aldrich®, Schnelldorf, Germany) with subsequent purification using QIAquick® PCR Purification Kit (QIAGEN, Hilden, Germany), according to the manufacturer’s instructions. Expression levels were evaluated using TaqMan® Gene Expression Assays (Applied Biosystems, Foster City, CA, USA), and *GUSB* was used as a reference gene for normalization, according to the formula: Relative expression = (Target gene mean quantity/Reference gene mean quantity). Ratios were then multiplied by 1,000 for easier tabulation. Each plate included multiple non-template controls and serial dilutions (10×) of a cDNA obtained from human prostate RNA (Ambion) were used to construct a standard curve for each plate. All experiments were run in triplicates.

### Luciferase assay

A reporter construct containing a binding site at *CCND2* 3′UTR for miR-375 (GeneCopoeia, Rockville, MD, USA) was introduced into PC-3 cells using Turbofectin 8.0 transfection reagent (Origene, Rockville, MD, USA). A vector without *CCND2* 3′UTR (GeneCopoeia) was used as experiment control. Vectors were co-transfected along with pre-miR-375 as described. Luciferase activity was assessed with the Secrete-Pair™ Dual Luminescence Assay Kit (GeneCopoeia, Rockville, MD, USA) according to the manufacturer’s instructions. Experiments were performed in triplicates at 48 and 72 h following co-transfection. At 72 h, miR-375 levels were measured by RT-qPCR to confirm its forced or silenced expression.

### TCGA data meta-analysis in prostate cancer patients

TCGA was used to obtain data on miRNA expression and clinical information, when available, from PCa and matched normal tissue samples [40]. All miRNA expression data from samples hybridized by the University of North Carolina, Lineberger Comprehensive Cancer Center, using Illumina HiSeq 2000 miRNA Sequencing, were downloaded from TCGA data matrix (http://tcga-data.nci.nih.gov/tcga/tcgaDownload.jsp). This dataset included 326 PCa and 50 matched normal patient samples. To prevent duplicates, when there was more than one portion per patient, median values were used. The provided value was pre-processed and normalized according to ‘level 3’ specifications of TCGA (see http://cancergenome.nih.gov/ for details). Clinical data of each patient was provided by the Biospecimen Core Resources (BCRs). This data is available for download through TCGA data matrix (http://tcga-data.nci.nih.gov/tcga/dataAccessMatrix.htm).

### Statistical analysis

The Shapiro-Wilk’s *W* test allowed for the examination of the appropriateness of a normal distribution assumption for each of the parameters (data not shown). Comparisons between two groups were then performed using non-parametric Mann–Whitney *U*-test. *P* values were considered statistically significant if lower than 0.05.

Correlation between miRNAs’ expression was measured by the Spearman’s correlation coefficient (*r*). Differences in miR-375 expression between N0 and N1 lymph node stage groups were assessed by Student’s *t*-test.

Statistical analysis was performed SPSS 20.0 for Mac (IBM-SPSS Inc., Chicago, IL, USA), and graphs were built using GraphPad Prism 5.0 software for Mac (GraphPad Software Inc., La Jolla, CA, USA).

## Additional files

Additional file 1: Table S1. Clinical and pathological data of patients included in this study for miR-32 and miR-182. Additional file 2: Figure S1. Validation of expression levels of (A) miR-32, (B) miR-182 (****P* < 0.001; ns, non-significant). Additional file 3: Figure S4. Expression of miR-32 and miR-182 is increased in prostate cancer in patients from TCGA. Additional file 4: Figure S2. Invasion assay in PC-3 cell line. Representative display of (A) miR-NC cells and (B) pre-miR-375 50 nM transfected cells. Additional file 5: Figure S3. Potential miR-375 target cancer-related genes in 22Rv1 and PC-3 transfected cells, normalized to miR-NC.
