# Supplementary material for: MicroRNA-375 plays a dual role in prostate carcinogenesis
Source: Clin Epigenetics. 2015 Apr 10;7(1):42. doi: 10.1186/s13148-015-0076-2 (PMC4431534; doi:10.1186/s13148-015-0076-2)
Supplement: Additional file 1: Table S1. — Clinical and pathological data of patients included in this study for miR-32 and miR-182. [file 13148_2015_76_MOESM1_ESM.doc]

Additional file 1: Table S1Clinical and pathological data of patients included in this study for miR-32 and miR-182.

| **Clinicopathological Data** | **Tumors**  **(n= 80)** | **MNPT**  **(n= 15)** |
| --- | --- | --- |
| **Age (yrs),** median (range) | 65 (49-74) | 64 (45-80) |
| PSA (ng/mL), median (range) | 8.00(2.7-20.0) | n.a. |
| **Pathological Stage, n (%)** | | |
| **pT2** | 49 (61.3%) | n.a. |
| **pT3a** | 24 (30.0%) | n.a. |
| **pT3b** | 7 (8.7%) | n.a. |
| **Gleason Score n (%)** | | |
| **< 7** | 25 (31.3%) | n.a. |
| **= 7** | 47 (58.7%) | n.a. |
| **> 7** | 8 (10.0%) | n.a. |

n.a., not applicable.
