# Supplementary figures and images for: MicroRNA-375 plays a dual role in prostate carcinogenesis
Source: Clin Epigenetics. 2015 Apr 10;7(1):42. doi: 10.1186/s13148-015-0076-2 (PMC4431534; doi:10.1186/s13148-015-0076-2)

**A****miR-32**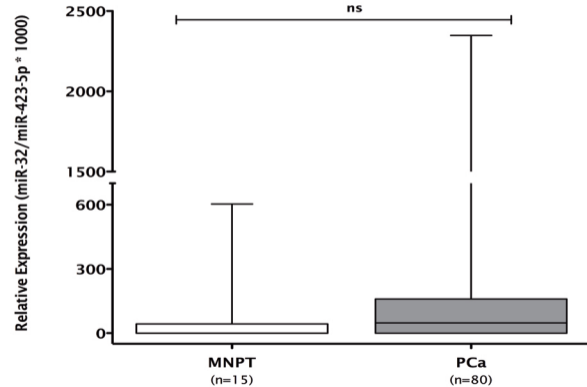**B****miR-182**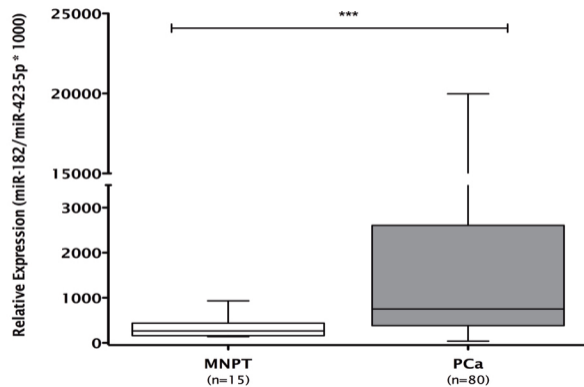

Supplement: Additional file 2: Figure S1. — Validation of expression levels of (A) miR-32, (B) miR-182 (***P < 0.001; ns, non-significant). [file 13148_2015_76_MOESM2_ESM.pdf]

**A**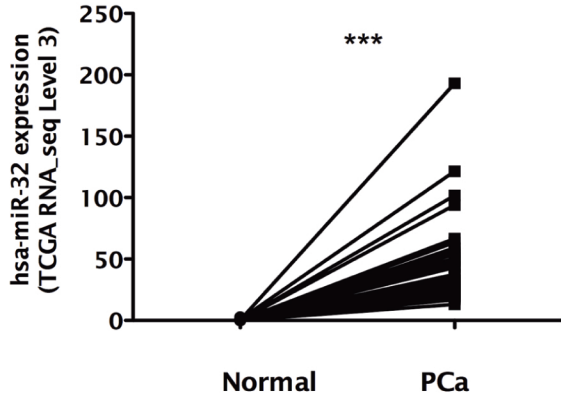**B**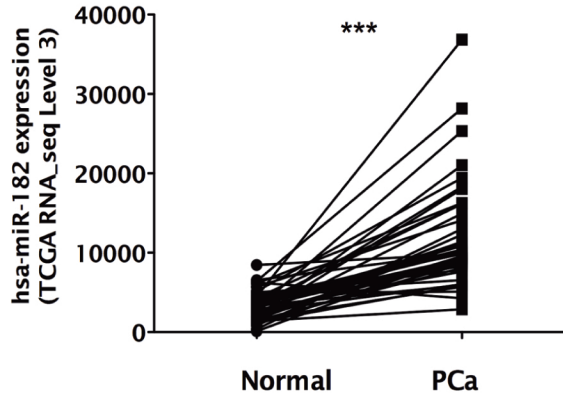

Supplement: Additional file 3: Figure S4. — Expression of miR-32 and miR-182 is increased in prostate cancer in patients from TCGA. [file 13148_2015_76_MOESM3_ESM.pdf]

**A**

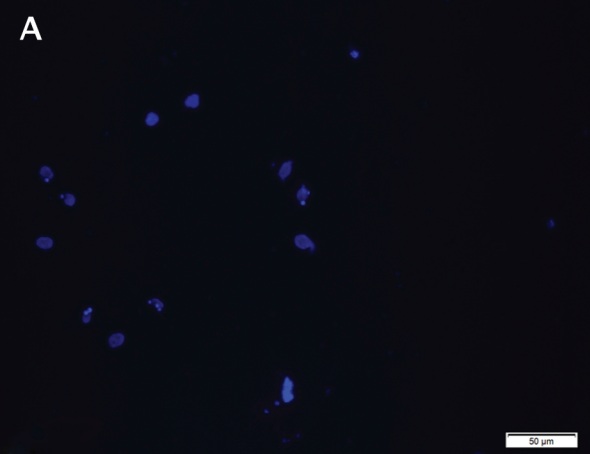

**B**

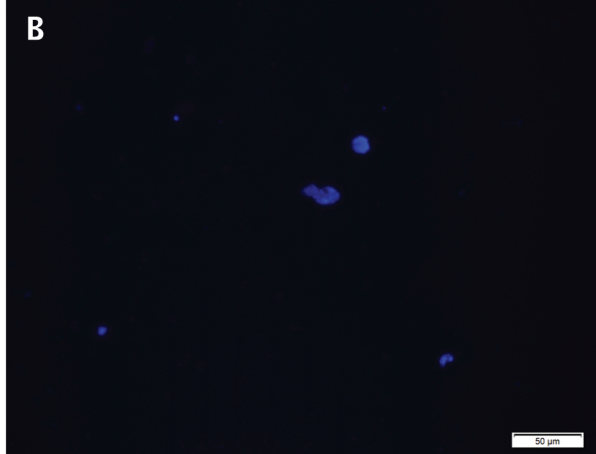

Supplement: Additional file 4: Figure S2. — Invasion assay in PC-3 cell line. Representative display of (A) miR-NC cells and (B) pre-miR-375 50 nM transfected cells. [file 13148_2015_76_MOESM4_ESM.pdf]

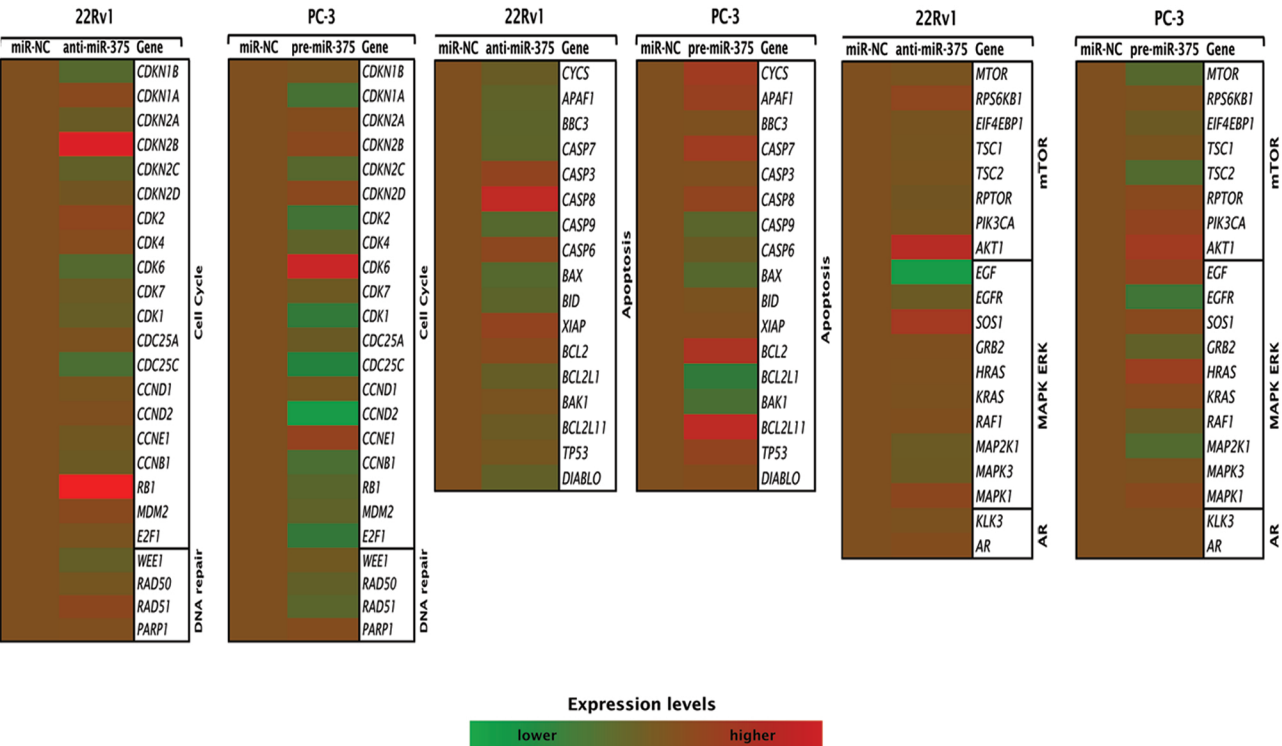

Supplement: Additional file 5: Figure S3. — Potential miR-375 target cancer-related genes in 22Rv1 and PC-3 transfected cells, normalized to miR-NC. [file 13148_2015_76_MOESM5_ESM.pdf]
